# Association between serum potassium levels and peritonitis in peritoneal dialysis patients: a longitudinal study

**DOI:** 10.1186/s12882-025-04690-3

**Published:** 2026-01-16

**Authors:** Weixin Tang, Shaojie Zhang, Wanyu Yang, Lichun Hong, Lin Jiang, Xinyue Su, Junli Jin, Qijing Zhang, Xun Tang, Tingting Guo, Zirui Chen, Qi Ye, Yujing Zhou, Xiang Lv, Yu Zhang, Jun Zhang

**Affiliations:** 1 Department of Nephrology, Zhujiang Hospital of Southern Medical University, Guangzhou, China; 2 Intensive Care Unit, The Second Affiliated Hospital, School of Medicine, The Chinese University of Hong Kong, Shenzhen & Longgang District People’s Hospital of Shenzhen, Shenzhen, China; 3 Department of Computer Science and Engineering, Southern University of Science and Technology, Shenzhen, China; 4 Peng Cheng Laboratory, Shenzhen, China

**Keywords:** Longitudinal serum potassium, Peritoneal dialysis, Peritonitis

## Abstract

**Background:**

Abnormal serum potassium levels are common among peritoneal dialysis (PD) patients. Many studies have shown hypokalemia as a risk factor for peritonitis, but most were cross-sectional and observational. We intended to analyze the longitudinal association between serum potassium levels and peritonitis in those undergoing PD.

**Methods:**

We included 1,288 patients undergoing regular PD at our institution. The endpoint event was peritonitis. Patients were divided into peritonitis and non-peritonitis groups. The relationship between baseline data and the emergence of peritonitis was analyzed through Cox regression analysis. Mixed-effects model was used to analyze the correlation between longitudinal serum potassium and other lab characteristics with peritonitis. Kaplan-Meier survival analysis estimated the median time to peritonitis.Independent samples t-test was used in subgroup analysis to explore the relationship between serum potassium and different pathogenic bacteria. Spearman correlation analysis and scatter plot were used to evaluate the correlation between serum potassium and magnesium. Cochran-Armitage trend chi-square test assessed the trend of peritonitis incidence.

**Results:**

COX regression analysis found higher baseline lymphocyte count and female gender were associated with lower peritonitis risk, while older age and higher baseline uric acid levels were linked to higher risk. A mixed-effects model indicated that the peritonitis group’s serum potassium decreased more rapidly and remained low longer. Kaplan-Meier curves estimated the median time to peritonitis to be 4.09 years. The analysis of subgroups found no significant difference in serum potassium levels between the gram-positive and gram-negative bacteria groups. Spearman correlation analysis showed a very weak positive correlation between potassium and magnesium with poor trend consistency but statistical significance. Peritonitis incidence showed a significant linear downward trend from 2011 to 2023.

**Conclusions:**

Rapid declines and long-term low levels of serum potassium after PD initiation increase peritonitis risk. Long-term potassium management in PD patients is crucial in clinic practice, with intensified monitoring advised around 4 years into PD treatment.

**Trial registration:**

2023BA0125_GC; 2023-10-20.

## Background

Recent studies show that the prevalence of chronic kidney diseases (CKD) among adults in China is 8.2% (95% CI, 7.8%-8.6%), which is higher than the global average [1]. As a replacement therapy, PD is a vital for patients with end-stage renal disease (ESRD), but long-term PD can lead to complications that may be life-threatening and affect patient prognosis [2]. Peritoneal dialysis-associated peritonitis (PDAP) is one of the most common and serious complications of PD, with treatment failure rates reported as high as 25% [3]. Although advances in PD connectivity and other evidence-based prevention strategies have reduced the incidence of peritonitis, peritonitis remains a major cause of conversion to hemodialysis (HD) and increases patient mortality [4, 5]. Some risk factors of peritonitis have been identified, but many of these are not modifiable. One risk factor that deserves closer scrutiny is hypokalemia. Hypokalemia is a common electrolyte abnormality in PD patients and has been associated with increased risks of peritonitis and death [6, 7]. Previous studies were largely cross-sectional and observational, with few addressing the dynamic changes in serum potassium. As a result, the precise relationship between hypokalemia and peritonitis remains uncertain. This study aims to investigate the relationship between fluctuations in longitudinal serum potassium levels and the risk of PDAP. Additionally, it analyzes the other risk factors associated with PDAP in PD patients.

## Methods

### Study subjects

**Fig. 1:**
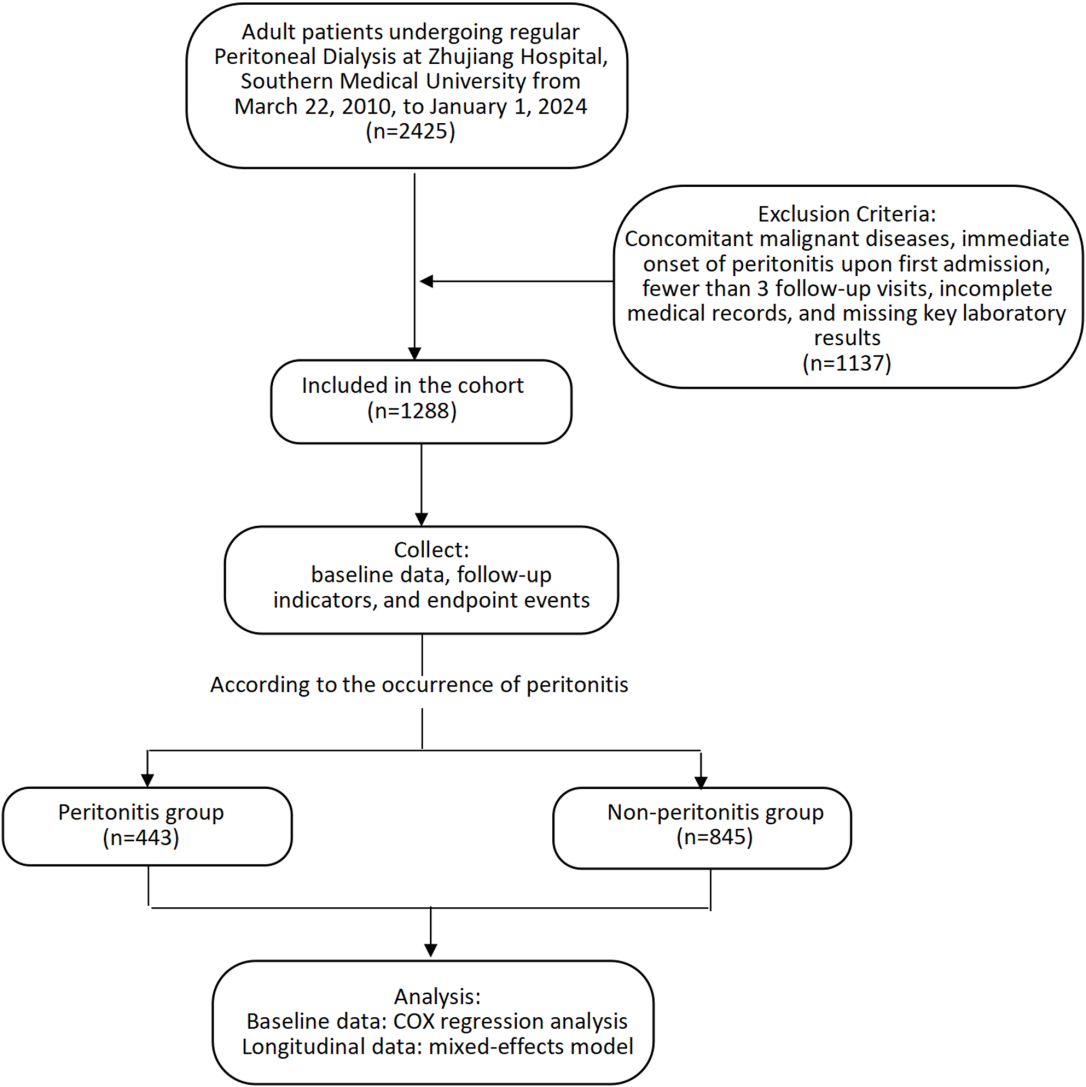
Flowchart of patient screening

This retrospective observational study included 2,425 patients undergoing regular PD at the Nephrology Department of Zhujiang Hospital of Southern Medical University from March 22, 2010, to January 1, 2024. Patients who developed peritonitis during the follow-up period had their first peritonitis episode recorded as the endpoint event. For patients who did not develop peritonitis, the date of the last follow-up was used as the outcome date. The patient screening flowchart is shown in Fig. 1. Medical Ethics Committee of Zhujiang Hospital of Southern Medical University approved the study, and it strictly followed the ethical principles of the Declaration of Helsinki (2013 revision). The retrospective nature of the study allowed for a waiver of informed consent.

### Relevant definitions and inclusion and exclusion criteria

Inclusion Criteria: Adult PD patients hospitalized in our PD center from March 22, 2010, to January 1, 2024.

Exclusion Criteria: Concomitant malignant diseases, immediate onset of peritonitis upon first admission, fewer than three follow-up visits, incomplete medical records, and missing key laboratory results.

### Data collection

Patient data collected included general demographic information (sex, age, BMI, comorbid conditions such as hypertension, diabetes, heart failure, and stroke) and laboratory test results from per hospital admissions (serum potassium, transferrin, hemoglobin, platelets, creatinine, human serum albumin, white blood cells, absolute peripheral blood lymphocyte count, and serum uric acid levels).

### Study endpoint

The endpoint is PDAP which was diagnosed according to the International Society for Peritoneal Dialysis (ISPD) guidelines [8], when met at least two of the following criteria: (1) clinical symptoms and signs of peritonitis, such as abdominal pain and/or cloudy effluent; (2) effluent white blood cell count >100 cells/µL with >50% neutrophils; (3) positive culture of peritoneal effluent. Diagnosis requires meeting any two of these three criteria. Patients were divided into two groups according to whether peritonitis occurred as Peritonitis Group and Non-Peritonitis Group.

### Statistical methods

Statistical analysis was conducted using R (version 4.3.0) and SPSS 27. Continuous data are expressed as mean ± standard deviation (SD) and compared using the Wilcoxon rank-sum test. Categorical data are expressed as frequency (percentage) and compared using Pearson’s Chi-squared test. Cox proportional hazards models were fitted using the survival package (version 3.6.4), and Kaplan-Meier curves were plotted using the survminer package (version 0.4.9). Mixed-effects models were fitted using the lme4 package (version 1.1–35.2), with p-values for model parameter tests obtained using the lmerTest package (version 3.1-3). An independent samples t-test was applied for the comparison of measurement data between the two groups. Trends over time among groups were illustrated via predictive plots generated using the ggeffects package (version 1.7.0). Spearman analysis and scatter plot were used for cohort analysis of continuous variables. Cochran-Armitage Trend Test was used to analyze the linear trend between ordinal categorical variables and binary outcome variables. A p-value of < 0.05 was considered statistically significant.

## Results

### Patient characteristics

A total of 1,288 regular PD patients were included based on inclusion and exclusion criteria, with an average age of 57.12 ± 14.51 years; 785 were male (60.79%) and 505 were female (39.21%); 953 had hypertension (73.99%), 363 had diabetes (28.18%), 204 had heart failure (15.84%), and 85 had a history of stroke (6.60%). At the end of follow-up, patients were divided into two groups: 443 in the peritonitis group and 845 in the non-peritonitis group. Statistical comparison revealed that age and the prevalence of hypertension and heart failure differed significantly between the two groups (*P* < 0.05). Additionally, baseline levels of creatinine, albumin, absolute lymphocyte count, and uric acid showed significant differences (*P* < 0.05) between groups, while sex, BMI, diabetes, stroke, serum potassium, transferrin, hemoglobin, platelet count, and white blood cell count did not (*P* > 0.05) (Table 1).

**Table 1:** Baseline characteristics of PD patients in the non-peritonitis group and peritonitis group

Variables	Total^1^ (*n* = 1288)	Non-peritonitis^1^ (*n* = 845)	Peritonitis^1^ (*n* = 443)	*P*-Value^2^
Gender				0.078
Male	783(60.79%)	499(59%)	284(64%)	
Female	505(39.21%)	346(41%)	159(36%)	
Age, years	57.13 ± 1 4.52	56 ± 15	59 ± 13	<0.001
BMI, kg/m^2^	22.49 (20.45,24.97)	22.8 ± 3.7 | 22.4 (20.3,25.2)	22.8 ± 3.6 | 22.6 (20.7,24.9)	0.6
Comorbidities				
Hypertension	953 (73.99%)	649 (77%)	304 (69%)	0.001
Diabetes	363 (28.18%)	250 (30%)	113 (26%)	0.12
Heart failure	204 (15.84%)	147 (17%)	57 (13%)	0.034
Stroke	85 (6.60%)	63 (7.5%)	22 (5.0%)	0.087
Baseline Laboratory Indicators				
Serum potassium, mmol/L	5.37 ± 0.28	5.37 ± 0.28	5.37 ± 0.28	0.50
Transferrin, g/L	1.77 ± 0.40	1.79 ± 0.41	1.73 ± 0.38	0.10
Hemoglobin, g/L	82.84 ± 19.63	82 ± 20	84 ± 20	0.15
Platelet count, ×10^9^/L	209.84 ± 81.88	213 ± 83	203 ± 79	0.05
Creatinine, µmol/L	963.08 ± 398.51	951 ± 396	987 ± 404	0.03
Human serum albumin, g/L	34.46 ± 5.90	34.2 ± 5.9	34.9 ± 5.8	0.02
White blood cell count, ×10^9^/L	7.36 ± 2.96	7.43 ± 3.03	7.20 ± 2.80	0.12
Absolute lymphocyte count, ×10^9^/L	1.31 ± 0.58	1.33 ± 0.59	1.26 ± 0.55	0.03
Uric acid, µmol/L	523.28 ± 144.29	513 ± 145	544 ± 140	< 0.001

^1^n (%); Mean ± SD | Median (IQR)

^2^Pearson’s Chi-squared test; Wilcoxon rank sum test; Fisher’s exact test

### COX regression analysis of baseline data and peritonitis

**Fig. 2:**
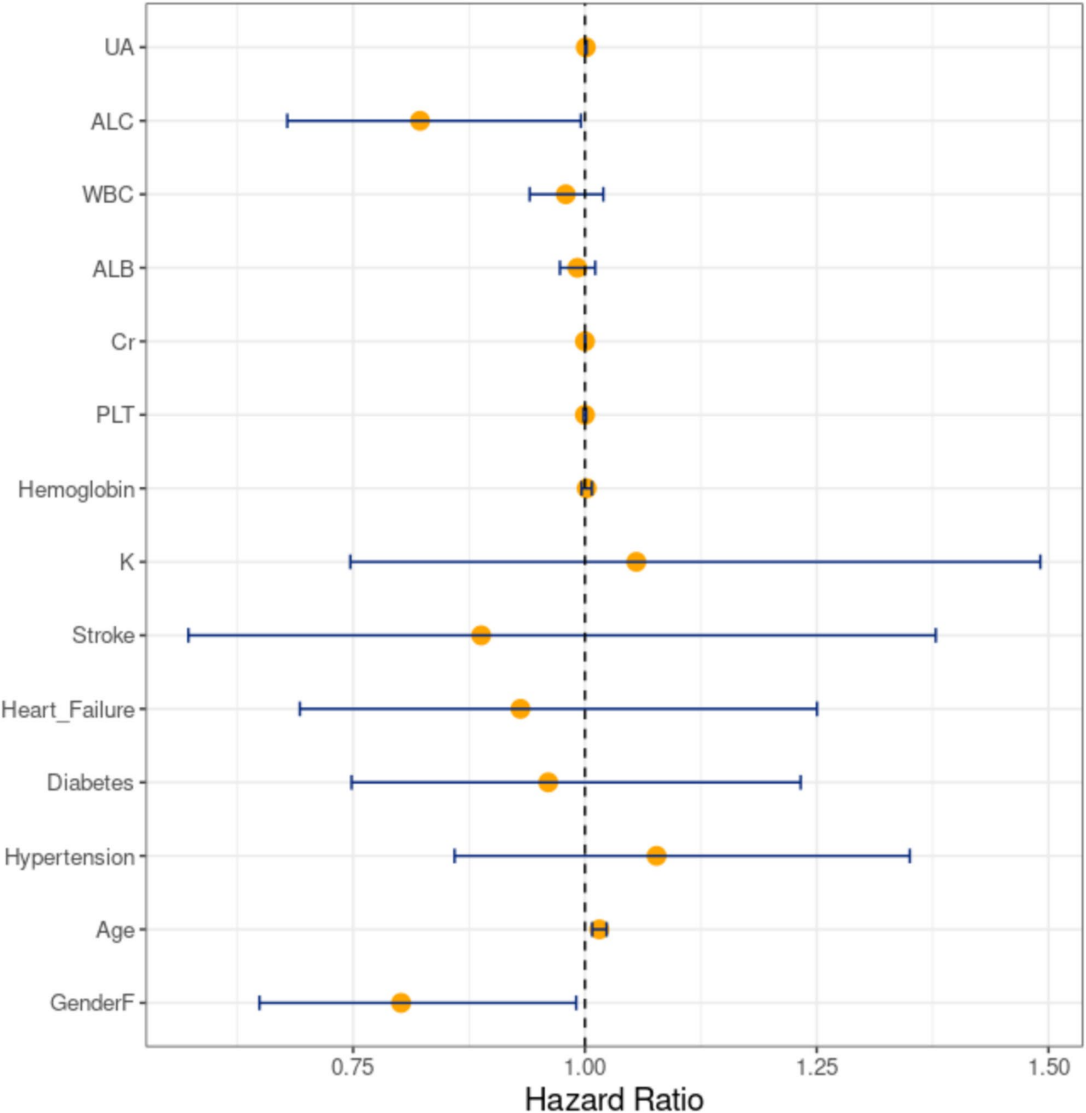
Forest plot of baseline characteristics and the risk of peritonitis

Cox proportional hazards models were used to analyze the relationship between baseline data and the occurrence of peritonitis, and a forest plot was created to illustrate these relationships (Fig. 2). The analysis indicated that higher absolute peripheral lymphocyte count (HR = 0.822), female sex (HR = 0.802), older age (HR = 1.016), and higher uric acid levels (HR = 1.001) were significantly associated with peritonitis occurrence (*P* < 0.05). Other baseline indicators showed no significant differences between groups.

### Analysis of follow-up test index changes and peritonitis in PD patients

**Fig. 3:**
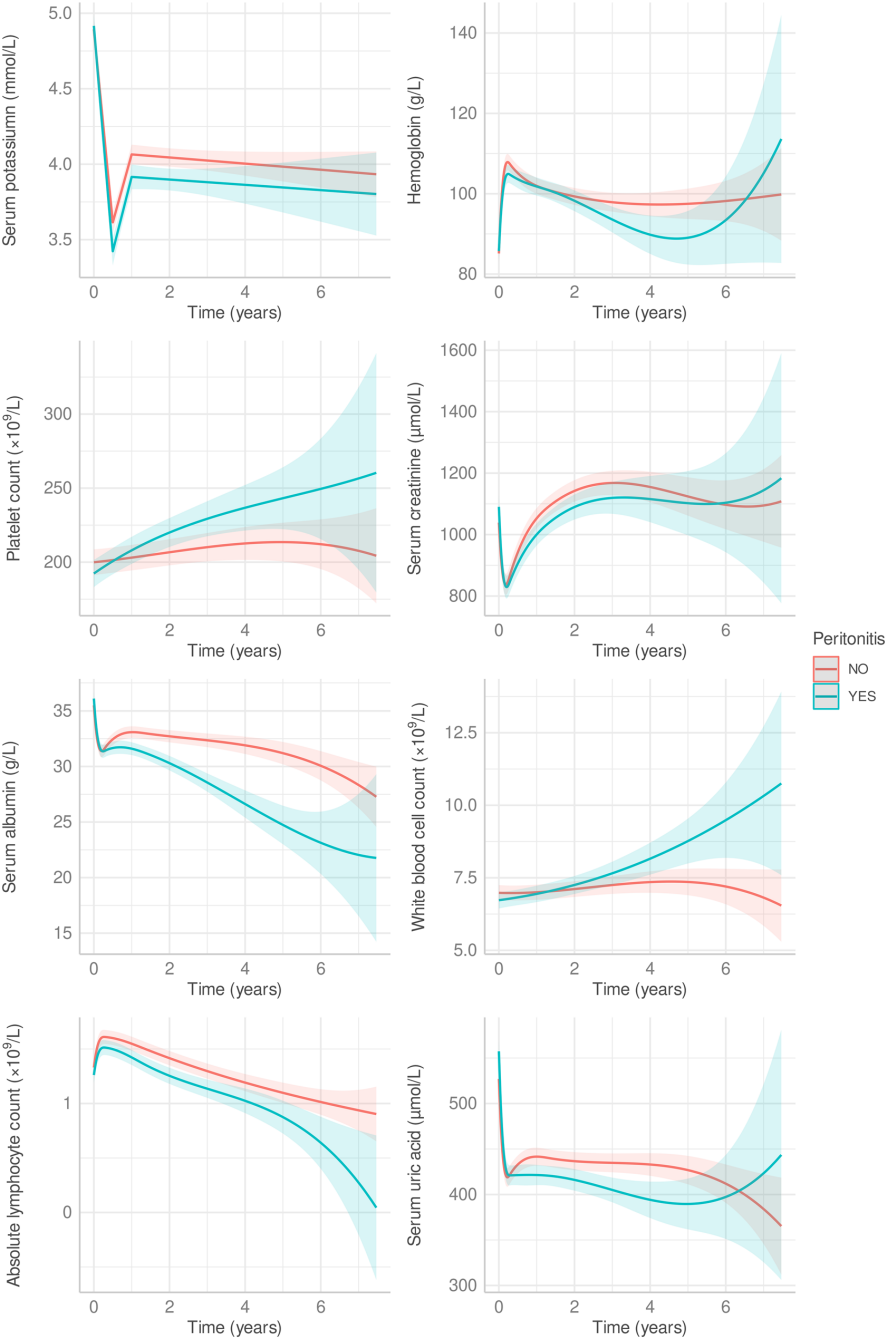
Mixed-effects model of the relationship between follow-up laboratory indicators and PDAP

Patients who met the screening criteria were analyzed, and baseline variables such as age and sex were included. Measurements from various hospital admissions within 7.5 years of the initial admission were recorded. Time was treated as a continuous variable in years, and patients were divided into peritonitis and non-peritonitis groups. The study aimed to explore whether data trends differed over time between the two groups. Results revealed that although there were no statistically significant differences in initial baseline potassium levels between the two groups, the peritonitis group experienced a more rapid decline in serum potassium levels, which remained significantly lower than the non-peritonitis group during follow-up. Albumin and absolute lymphocyte count trends were also consistently lower in the peritonitis group (Fig. 3).

### Median time to peritonitis in PD patients

**Fig. 4:**
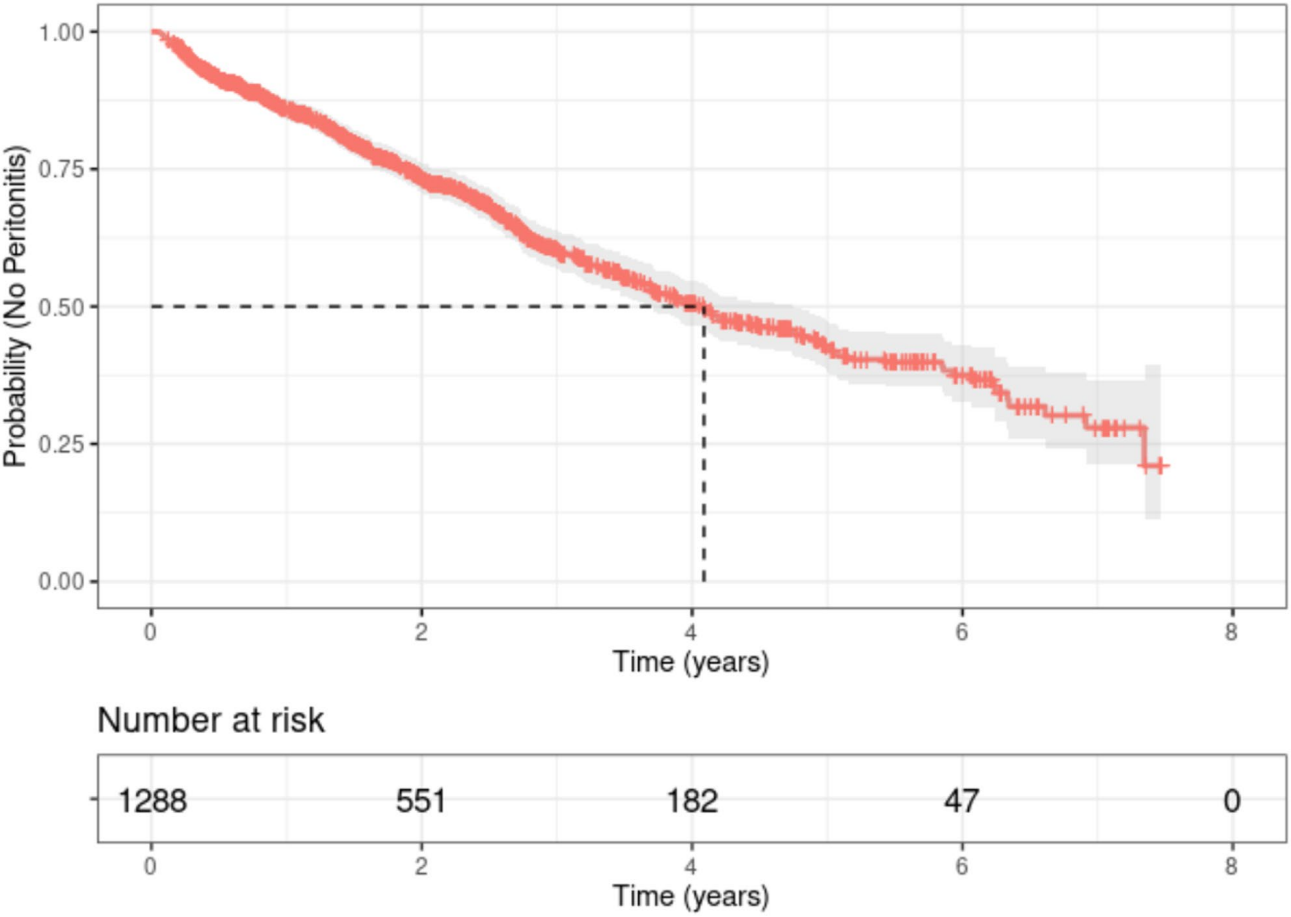
Kaplan-Meier plot for PDAP

For all included patients (*n* = 1288), the date of initial PD admission was recorded, and for the peritonitis group, the date of first peritonitis occurrence was additionally recorded. Kaplan-Meier survival curves estimated the median time from the initiation of PD treatment to the development of peritonitis was 4.09 years (95% CI: 3.7–4.74) (Fig. 4), indicating a 50% risk of peritonitis 4.09 years into PD treatment.

### Correlation between pathogenic microorganism types of PDAP and serum potassium

**Fig. 5:**
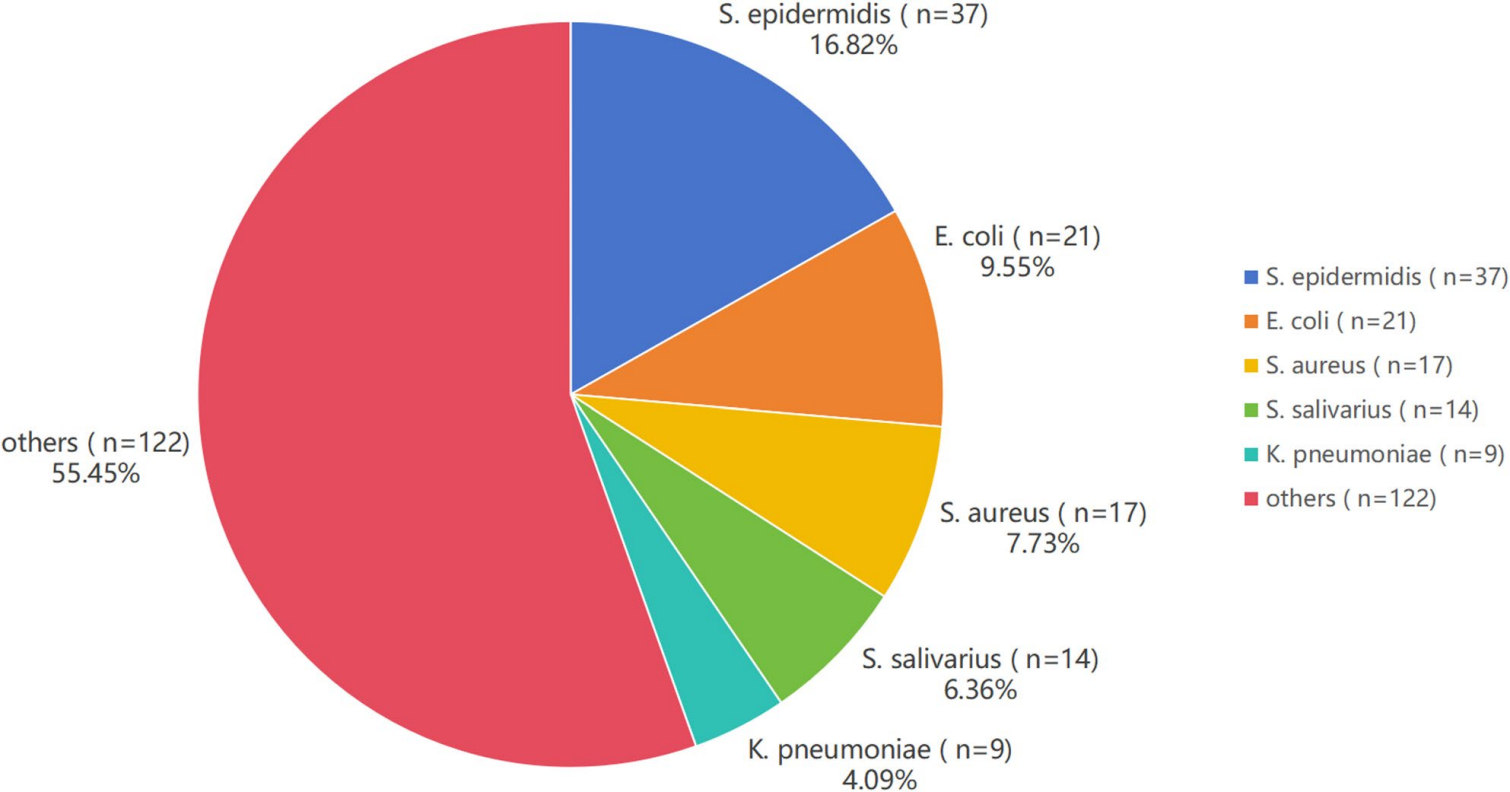
Relative abundance of major bacterial species (%)

Among 443 patients in the peritonitis group, 220 had positive pathogenic culture results. Gram-positive cocci (GPC) were the most common pathogens (*n* = 140, 63.64%), followed by Gram-negative bacilli (GNB) (*n* = 71, 32.27%), fungi (*n* = 7, 3.18%), and mixed infections (*n* = 2, 0.91%). The top five pathogenic microorganisms were *Staphylococcus epidermidis*,* Escherichia coli*,* Staphylococcus aureus*,* Streptococcus salivarius*, and *Klebsiella pneumoniae* (Fig. 5).

Serum potassium levels were compared between GPC and GNB groups. The mean serum potassium was 4.77 (SD = 1.05) in the GPC group and 4.62 (SD = 0.86) in the GNB group (Table 2). Shapiro-Wilk tests indicated normality distribution in the GPC group (W = 0.956, *P* < 0.001) and normal distribution in the GNB group (W = 0.980, *P* = 0.303). Lilliefors-corrected Kolmogorov-Smirnov test showed consistent trends: *P* = 0.057 in the GPC group (approaching the critical value for normality) and *P* = 0.200 in the GNB group (normal distribution).

Levene’s test indicated homogeneity of variance (*P* = 0.207), so an independent samples t-test was performed. No major difference in serum potassium was observed between GPC and GNB groups (*P* = 0.295) (Table 3).

**Table 2:** Descriptive statistics of serum potassium between the two groups

Group	*n*	Mean	SD	SE
GPC	140	4.768	1.050	0.089
GNB	71	4.617	0.862	1.023

**Table 3:** Results of independent-samples t-test for serum potassium between the two groups

Levene’s Test	F	*P*	t-Test (Equal Variances Assumed)	t	df	*p* (two-tailed)	Mean Difference	95% CI (Difference)	Cohen’s d
Equal Variances	1.602	0.207		1.050	209	0.295	0.152	-0.133 to 0.436	0.153

### Correlation analysis of serum potassium and magnesium trends in PD patients

**Fig. 6:**
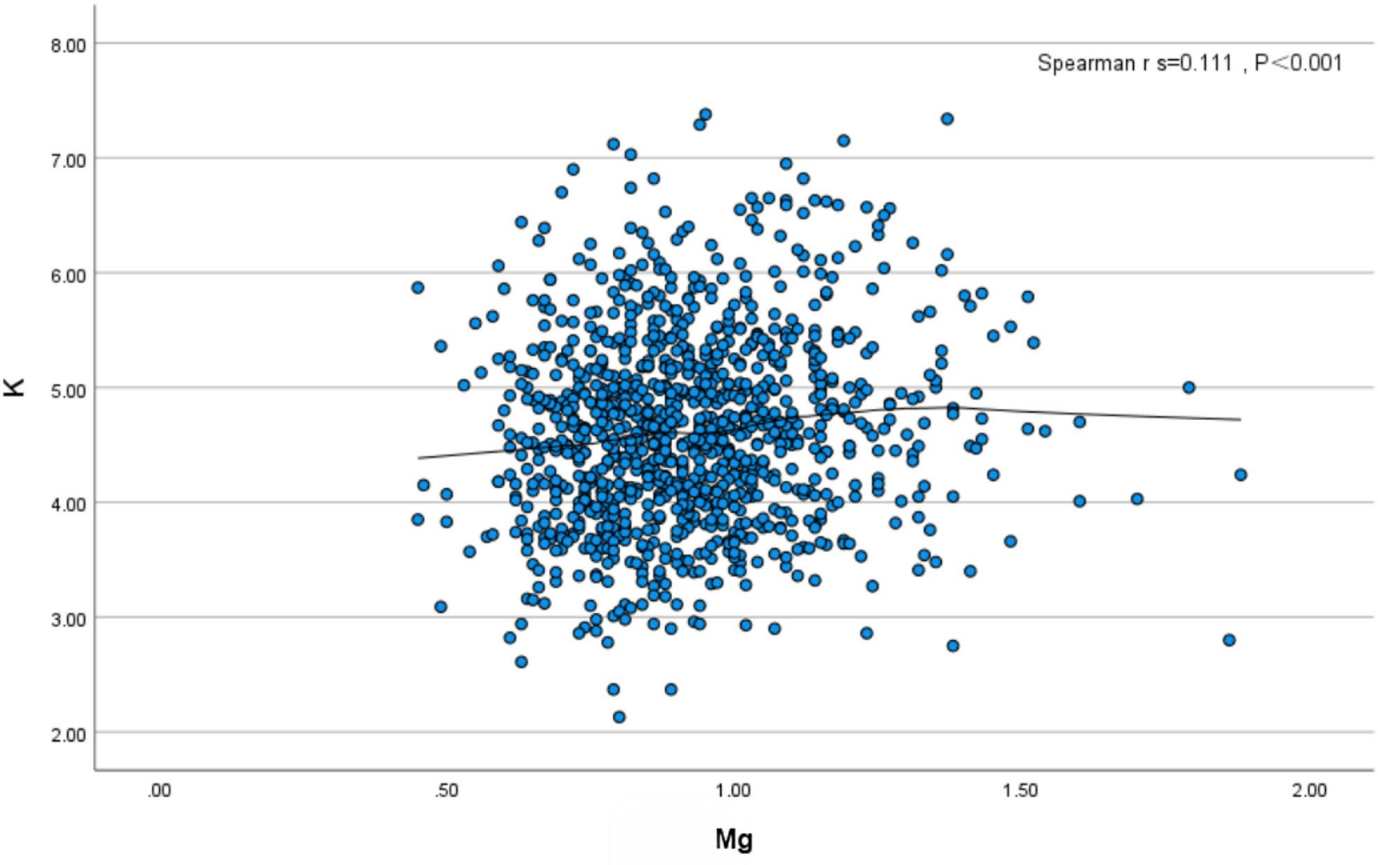
Scatter diagram of correlation between potassium and magnesium changes

Serum potassium and magnesium levels during follow-up were recorded for all included patients (*n* = 1288). Paired analysis was performed on simultaneous potassium and magnesium measurements from the same patient; single measurements where only one of the two ions (potassium or magnesium) was measured were excluded. Finally, 1032 pairs of simultaneous measurements were included in Spearman correlation analysis, and a scatter plot was generated (X-axis = Mg, Y-axis = K) (Fig. 6).

Spearman correlation analysis showed a very weak positive correlation between potassium and magnesium (rₛ=0.111, *P* < 0.01). The scatter points were distributed without a clear “bottom-left to top-right” trend (only a slight positive trend), which indicates a statistically significant but weak correlation, with poor consistency in the overall trend.

### Trend analysis of annual PDAP incidence

**Fig. 7:**
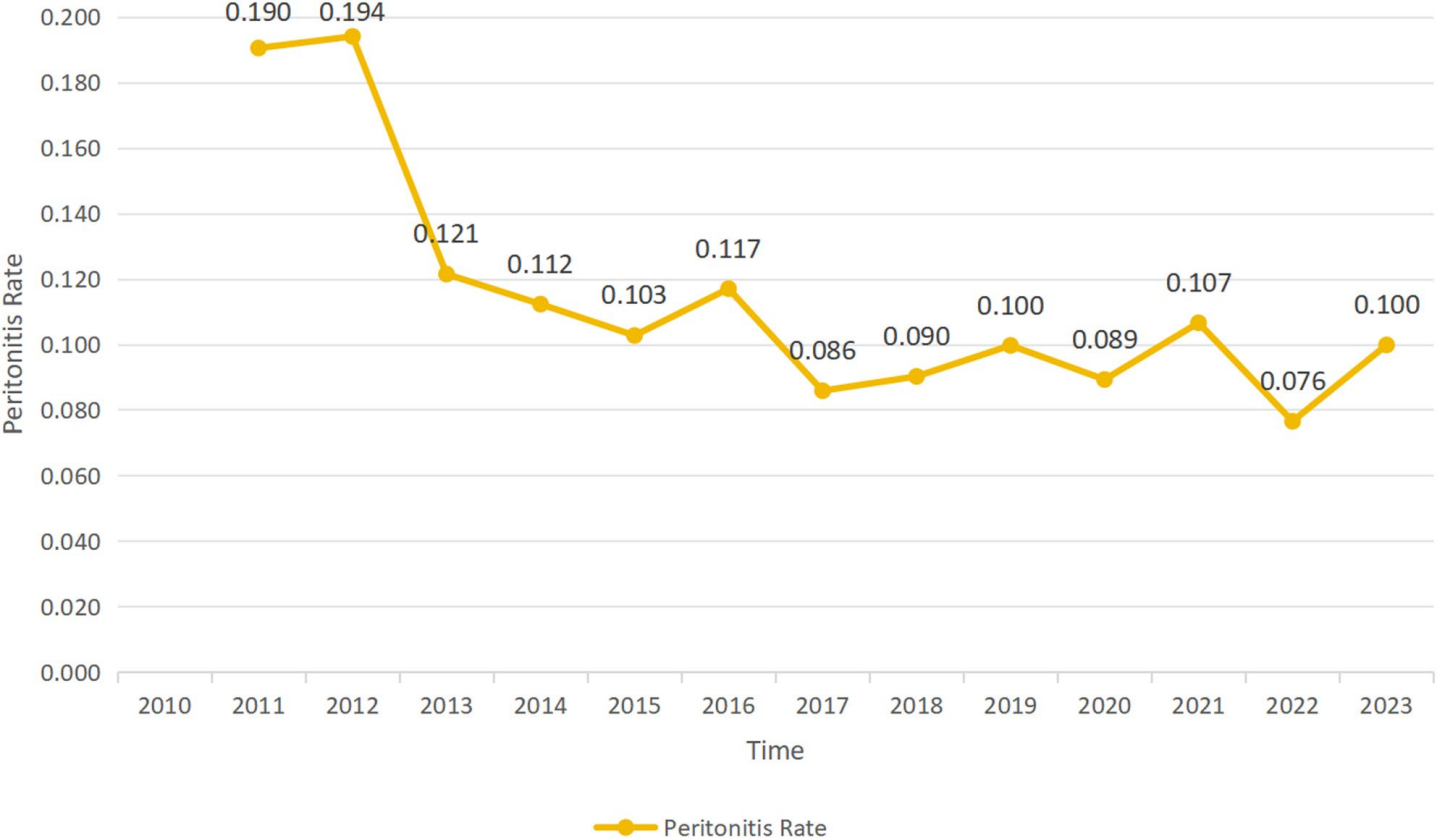
Line chart of annual trends in PDAP incidence (episode/patient year)

The number of regular PD patients and annual peritonitis cases were collected to calculate annual PDAP incidence, and a line chart was generated (Fig. 7). Data for 2010 were excluded due to small sample size (5 PD patients, 0 PDAP cases). The line chart showed two stages: rapid decline (2012–2017) and stable range with minor fluctuations (2018–2023). The overall PDAP incidence (episodes per patient-year) decreased by 47.6%, from 0.194 in 2011 to 0.100 in 2023. Cochran-Armitage trend test showed a significant linear downward trend in PDAP incidence from 2011 to 2023 (χ^2^ = 4.874, df = 1, *p* = 0.027 < 0.05).

## Discussion

Analysis of the Global Burden of Disease 2016 dataset shows an 87% rise in the global burden of chronic kidney disease and a doubling of chronic kidney disease deaths between 1990 and 2016 [9]. CKD has become an important public health problem in China [10]. Previous surveys indicate a growing number of CKD patients undergoing PD treatment, with [11]. PD is widely used clinically due to its better overall survival rate and quality of life, longer preservation of residual renal function, lower delayed graft function rate, and lower econoover 270,000 patients worldwide, accounting for approximately 11% of all dialysis patients mic cost [12]. However, long-term PD can lead to various complications, with approximately 16% of patients discontinuing PD or dying directly due to PDAP [13]. The ISPD has emphasized timely treatment for PDAP [8]. Despite increasing attention to PDAP, delayed medical visits still occur [14], negatively impacting patient prognosis. Therefore, identifying indicators that can effectively predict PDAP and are easy to monitor, simple, and cost-effective has become a recent focus.

Potassium is an essential electrolyte involved in cellular metabolism, closely related to glycogen and protein synthesis. It acts as an activator and cofactor for many enzymes, maintaining cell growth and acid-base balance, ensuring normal enzyme system function, and aiding DNA synthesis. Normal serum potassium concentration is 3.5-5.0 mmol/L, influenced by dietary potassium, renal function, medication, and acid-base status, and primarily excreted by the kidneys [15]. Kovesdy et al. [16] analyzed 27 international cohort studies using meta-analysis to assess the relationship between adverse outcomes and serum potassium levels in CKD patients. Results showed that a serum potassium level of 4.0-4.5 mmol/L had the lowest mortality risk, while levels outside the 3.5-5.0 mmol/L range had higher mortality risks [16–18]. Torlén et al. [19] confirmed that patients undergoing maintenance dialysis with serum potassium < 3.5 mmol/L had a higher risk of infection-related mortality. And they found that the impact of pre-dialysis hypokalemia on prognosis is related to inflammation, nutritional status, and comorbid conditions such as diabetes and cardiovascular disease [19]. Bielecka-Dabrowa et al. [20] demonstrated that advanced age, comorbid diabetes, cardiovascular diseases, and inflammation are risk factors for poor prognosis associated with abnormal serum potassium levels. Domestic studies [21] indicate that long-term fluctuations in serum potassium levels may correlate with poor outcomes in dialysis patients. Recent studies by Engelhardt et al. [22] and Lombardi et al. [23] consistently reported that significant fluctuations in serum potassium might increase the risk of arrhythmia-related death due to rapid changes in cell membrane resting potential, reflecting unstable conditions, and more severe underlying diseases, all of which can contribute to higher risks of peritonitis and death. Liu et al. [24] reported that a longer period of hypokalemia correlates with a higher incidence of peritonitis, with an increase of 1.2% and 4.6% for every extra month of hypokalemia and serious hypokalemia. When patients’ hypokalemia duration prolonged to more than 6 months, or serious hypokalemia duration of more than 3 months, the peritonitis incident greatly increases.

These data strongly suggested that a longer duration of hypokalemia might be even more impactful for peritonitis in PD patients. Hypokalemia in PD patients usually does not occur only once, and the long-term management of potassium in PD patients has received more and more attention. However, routine monitoring of post-dialysis serum potassium levels in PD patients is still rare, and studies on the impact of post-dialysis serum potassium levels on PD patient prognosis are limited, especially in China. Guidelines have not yet specified the optimal monitoring frequency for serum potassium, nor the ideal range for maintaining post-dialysis serum potassium to minimize PDAP risk. Our study highlights the importance of dynamic monitoring of serum potassium in peritoneal dialysis patients in clinic practice, especially at the beginning of peritoneal dialysis.

Analysis of a large sample revealed no significant correlation between initial baseline serum potassium levels and the subsequent occurrence of PDAP after starting PD. However, the rate of decline in serum potassium levels and their long-term maintenance levels significantly influenced PDAP occurrence. A faster decline and lower maintenance levels of serum potassium increased the risk of peritonitis. Additionally, the median time to PDAP among regular PD patients was approximately 4.09 years, suggesting that closer monitoring and preventive measures around this time could optimize resources, reduce PDAP incidence.

The relationship between hypokalemia and PDAP pathogen types is another important clinical research direction. Previous studies proposed potential mechanisms by which hypokalemia increases infection risk in PD patients: (1) Impaired immune function: Potassium is essential for maintaining normal cellular function, including immune cell activity. Hypokalemia can impair the respiratory burst function of neutrophils and monocytes, thereby reducing bacterial clearance and increasing infection susceptibility [25]. (2) Compromised nutritional status: Hypokalemia is associated with malnutrition and protein-energy wasting in PD patients [26, 27]. Malnutrition in turn further impairs immune function, reducing resistance to infection. (3) Altered intestinal motility: Potassium is critical for intestinal smooth muscle function. Severe hypokalemia causes reduced intestinal peristalsis or paralytic ileus, increasing intestinal bacterial overgrowth and translocation, thereby facilitating intraperitoneal bacterial invasion [28]. (4) Indirect effect on specific flora: Although no direct evidence links hypokalemia to specific flora, reduced immunity due to hypokalemia may increase the risk of infection by opportunistic pathogens (including GPC such as staphylococci and GNB such as *Escherichia coli*). Kovacevic et al. reported that GPC and GNB are common PDAP pathogens (*Staphylococcus aureus*,* coagulase-negative Staphylococcus*,* Streptococcus*,* Escherichia coli*,* Klebsiella*), with *Staphylococcus aureus* and GNB being major causes [29], consistent with our findings. Currently, most studies focus on the association between hypokalemia and overall peritonitis risk, with limited direct research on its impact on PDAP pathogen types. Our study explored this relationship but found no significant difference in serum potassium between GPC and GNB groups. Further microbiological and immunological studies are needed to clarify this relationship.

Previous studies confirmed that hypoalbuminemia is an independent risk factor for increased PDAP risk and plays an crucial role in PD patient prognosis [30–32]. Albumin is critical for maintaining osmotic pressure, substance transport, and immune regulation. Albumin loss in PD patients involves peritoneal ultrafiltration characteristics, permeability changes, inflammatory status, malnutrition, and dialysate biocompatibility [33, 34]. The relationship between hypoalbuminemia and PDAP involves multiple mechanisms: (1) Malnutrition is a common risk factor for both. PD patients often have chronic inflammation and malnutrition, reducing albumin synthesis and increasing its catabolism [34]. Impaired immune function in malnourished patients increases infection risk [35], and peritonitis further increases albumin loss, forming a vicious cycle [31]. (2) Albumin binds and clears endotoxins and free radicals, reducing inflammatory response and oxidative stress [36], so hypoalbuminemia may increase peritoneal susceptibility to bacterial invasion and inflammation [34]. (3) Poor dialysate biocompatibility contributes to peritoneal damage and albumin loss [37–39]: components such as high-glucose dialysate, low pH, lactic acid, and glucose degradation products induce mesothelial-mesenchymal transition and peritoneal fibrosis, impairing peritoneal barrier function and increasing transperitoneal albumin transport, which may lead to peritoneal failure.

Studies reported that low albumin levels at PD initiation or during PD can predict PDAP occurrence [30, 40]. Wang et al. [40] demonstrated that patients with low albumin levels at PD initiation had significantly higher peritonitis risk. Our study results were slightly different from these reports. Specifically, we found no significant difference in baseline albumin levels between the peritonitis and non-peritonitis groups (HR = 0.992, 95%CI: 0.973–1.011, *P*>0.05). However, during subsequent follow-up, albumin levels in the peritonitis group decreased significantly and remained consistently lower than those in the non-peritonitis group, with significant differences. In conclusion, there is a complex interaction between albumin levels and PDAP, which warrants further investigation in future studies.

This study also compared the changing trends of serum potassium and magnesium in long-term PD patients. Like potassium, magnesium is an important intracellular cation that plays a key role in maintaining membrane potential, neuromuscular excitability, and myocardial function [41], and is also involved in regulating the function of potassium channels in cell membranes. Hypomagnesemia can impair the intracellular storage and transmembrane distribution of potassium, potentially leading to intracellular potassium depletion despite normal serum potassium levels. Even when serum potassium levels are within the normal range, intracellular potassium deficiency may exist, thereby increasing the risk of arrhythmia. Both hypomagnesemia and hypokalemia are closely associated with poor prognosis in PD patients, and they may occur simultaneously due to common pathophysiological factors (such as dialysis clearance, insufficient diet) [42]. Although existing evidence fully indicates that the homeostasis of these two electrolytes is crucial for PD patients and they should be evaluated and managed as an interconnected entity in clinical management [43], there is currently a paucity of studies directly investigating the correlation between their temporal trends. This study found a very weak positive correlation between potassium and magnesium in long-term PD patients, and this association was statistically significant, providing a preliminary basis for optimizing electrolyte management in PD patients. As it is not the core research direction, further subgroup or multivariate analyses were not performed; thus, the observed correlation should be interpreted with caution due to potential confounding factors (e.g., nutritional status, medication use), and this correlation warrants detailed investigation in future studies.

Over the past decade, the incidence of PDAP in our center has decreased markedly, especially from 2012 to 2017, when it decreased from 0.194 to 0.086 episodes per patient-year (a total decrease of 55.77%). This should be the result of the combined effect of multiple factors: (1) Continuous update of management guidelines and refinement of management strategies: Clinicians have been able to diagnose peritonitis more accurately and take effective treatment measures in a timely manner (including rational antibiotic application, standardized intraperitoneal administration routes, and optimized pathogen monitoring). For example, the ISPD 2010 guidelines [44] provide standardized recommendations for the diagnosis, treatment, and prevention of peritonitis. A PD center in Sudan found that [45] the incidence of peritonitis decreased significantly in the 3 years before and after the implementation of ISPD standards, confirming that following and applying standardized PD management has a direct and strong role in lowering the incidence of peritonitis. (2) Continuous improvement of PD technology and medical staff skills: Advances in aseptic operation technology, optimization of catheter implantation technology, and improvement of exit site care have continuously improved the safety of PD patients [45–47]. Our center began sending nurses to study in PD specialist training programs in 2014 and introduced automated PD machines in 2017. These technological advancements are believed to contribute to the reduction in PDAP incidence. The ISPD 2022 guidelines recommend [48] that the overall peritonitis incidence should not exceed 0.40 episodes per patient-year (1 C). A seven-year study in two hospitals in Turkey [49] showed an average annual peritonitis incidence of 0.37 episodes per patient-year. A single-center clinical study in Italy from 2009 to 2023 reported [50] that the overall peritonitis rate was 0.25 episode per patient-year. According to an expert consensus by the Chinese Expert Group on Prevention and Treatment of Peritoneal Dialysis-related Infections [51], the peritonitis incidence reported by foreign peritoneal dialysis centers is mostly 0.06–1.66 episodes per patient-year, while that reported by large domestic peritoneal dialysis centers is 0.14–0.17 episodes per patient-year. Since 2018, the PDAP incidence in our center has remained at 0.08–0.10 episodes per patient-year, which is much lower than the levels at home and abroad, indicating good overall PD management currently. (3) Improvement of patient education and self-management ability: Continuous and regular retraining for PD patients is an important strategy for preventing peritonitis. A prospective randomized controlled trial in Beijing, China showed that regular retraining of patients on PD fluid exchange operations through technical inspections helps reduce the risk of non-enteric peritonitis [52]. To this end, our center has operated a PD nursing specialist clinic since 2015, providing targeted guidance and hands-on training for PD patients, which is also one of the factors contributing to the reduction in PDAP incidence. (4) Accumulation of medical practice and center experience: Through long-term experience accumulation, factors affecting PDAP can be identified, which undoubtedly helps improve the center’s management level and reduce the infection rate [53]. In summary, these factors promote each other and jointly promote the progress in the prevention and management of PDAP, effectively reducing the incidence of peritonitis.

### Limitations

This is a single-center retrospective study, and diet, medication, and other potential influencing factors were not included in the analysis, warranting further improvement. Nevertheless, dynamic monitoring of serum potassium levels during regular PD treatment can somewhat reflect patients’ condition characteristics at that time.

## Conclusions

Although the baseline serum potassium has no relation to the occurrence of peritonitis, the rate of decline and long-term maintenance levels of serum potassium after starting long-term PD affect the risk of peritonitis in Chinese maintenance PD patients, with hypokalemia associated with increased PDAP risk. Fluctuations in serum potassium can be a predictive and evaluative indicator for PDAP. Patients approaching 4 years of PD treatment should be considered to strengthen monitoring, with combined indicators of serum potassium levels and other risk factors aiding in peritonitis prediction. Implementing targeted measures through dynamic monitoring and stabilizing serum potassium levels can reduce PDAP incidence, improving PD success rates and patient prognosis, reducing medical costs, and offering significant social and economic benefits.

## Data Availability

The datasets generated and analyzed during the current study are not publicly available due ethical restrictions and participant confidentiality concerns but are available from the corresponding author to qualified researchers on reasonable request.

## Abbreviations

PD: Peritoneal dialysis
CKD: Chronic kidney diseases
ESRD: End-stage renal disease
PDAP: Peritoneal dialysis-associated peritonitis
HD: Hemodialysis
ISPD: International Society for Peritoneal Dialysis
SD: Standard deviation
GPC: Gram-Positive Cocci
GNB: Gram-Negative Bacilli
